# “This Is Not Gym”: Enacting Student Voice Pedagogies to Promote Social and Emotional Learning and Meaningful Physical Education

**DOI:** 10.3389/fspor.2021.764613

**Published:** 2021-10-26

**Authors:** Donal Howley, Ben Dyson, Seunghyun Baek, Judy Fowler, Yanhua Shen

**Affiliations:** Department of Kinesiology, University of North Carolina at Greensboro, Greensboro, NC, United States

**Keywords:** physical education, youth voice, social and emotional learning (SEL), meaningful physical education, student voice and participation, pedagogy, alternative education

## Abstract

The purpose of this study was to explore learners' experiences enacting youth/student voice pedagogies (SVP) to promote Social and Emotional Learning (SEL) and meaningful physical education (MPE) in an alternative education setting. Drawing on social constructivist learning theory in understanding and implementing a MPE approach, and a systemic framework for SEL, two research questions guided the research process: (1) How did students interpret and enact these pedagogies? (2) What contribution did the enactment of these pedagogies have in promoting SEL and MPE? This study implemented a qualitative case study design framed by a participatory action research (PAR) approach spanning 12 weeks from February to May 2021. Participants in this study included 16 ninth grade alternative high school students (eight girls/eight boys) aged 14–15 who had just returned to face-to-face learning in January 2021 for the first time following COVID-19. A range of traditional and innovative participatory qualitative research methods including focus group interviews, students' personal biographies, timelines, digital and written reflections, photovoice, and class artifacts were utilized. The *Miles, Huberman, and Saldana Framework for Qualitative Data Analysis* was implemented involving both deductive and inductive combinations of comparative and thematic analysis. The following themes were constructed: *Making responsible decisions; unearthing and sharing mixed emotions; picturing physical activity beyond the classroom; recognizing the role of relationships; considering challenge and competence;* and, *pursuing meaning*. Findings demonstrate how enacting SVP can lead to the development of students' SEL and MPE experiences complimenting multiple learning domains. We call for further embedding of SVP capturing students' physical activity and movement experiences inside and outside of PE in teacher education and professional development that helps teachers and their students make sense of, shape, influence, and enact more MPE and physical activity learning experiences.

## Introduction

Reflecting trends in education and societies more broadly (Gonzalez et al., 2017; Cook-Sather, 2018; Mitra, 2018; Mills et al., 2021), the last decade has seen considerable scaling up of research and advocacy for enacting youth/student voice within and across physical education (PE), physical activity, and youth sport settings (Hooper and Sandford, 2021; Iannucci and Parker, 2021). Encouragingly, such work is increasingly exploring the enactment of youth/student voice pedagogies (SVP) with historically disengaged, underserved, and marginalized youth also. Such populations include, but are not limited to, girls (Enright and O'Sullivan, 2010a; Oliver and Kirk, 2015, 2016; Gray et al., 2019), racial and ethnic minorities (Hamzeh and Oliver, 2012; Pang and Macdonald, 2016; Thorjussen and Sisjord, 2018; Safron, 2020), LGBTIQ+ students (Drury et al., 2017; Berg and Kokkonen, 2021; Safron and Landi, 2021), underserved (Ward and Parker, 2013; Luguetti et al., 2017a,b), disabled (Fitzgerald and Stride, 2012; Meegan, 2018; Apelmo, 2019; Maher and Haegele, 2021), individuals with neuro-development disorders (Lamb et al., 2016; Thoren et al., 2020), and care-experienced youth (Quarmby et al., 2019, 2021; Sandford et al., 2021). Such work has emphasized the need to enact more transformative, socially just, and democratic approaches to PE which cater for and enhance students' broader learning beyond PE subject matter (Lynch and Curtner-Smith, 2019).

At the same time, research simultaneously demonstrates the tensions and problematics which exist in enacting such an approach with young people (Glasby and Macdonald, 2004; Öhman and Quennerstedt, 2008; Azzarito, 2009, 2016; Howley and O'Sullivan, 2020, 2021). Indeed, when we consider PE settings specifically, the enactment of SVP has not yet truly manifested itself in contemporary teaching and learning practices globally (O'Sullivan, 2018; Fitzpatrick, 2019; Quennerstedt, 2019). What is often found instead, are research methodologies and pedagogies which capture the voice of students primarily as one-off data sources rather than continuous creators and responders having agency in such a process (Dyson, 2006; Lundy, 2007; Fitzgerald et al., 2021). Distinguishing genuine student voice from adult dominated research agendas is important in this regard. Any enactment claiming itself to encompass SVP must involve: (1) democratically grounded learner-centered pedagogies that allow participants to understand and take ownership and responsibility for their learning (Lynch and Curtner-Smith, 2019); (2) processes which create communities of learning, where collaboration and cooperation are the norm and students have opportunities to participate in decision making (Hytten, 2017); and (3) provide participants opportunities to share and reflect on their learning experiences while continuing to influence analyses, decisions, and practices (Cook-Sather, 2006, 2014). Such spaces require a deliberate focus on pedagogies that can accomplish a range of holistic outcomes through the teaching and of learning PE content. Despite the good work previously completed, the need to better elicit, understand, and work with young people in PE, physical activity, and youth sport settings continues. An area of increasingly growing interest in this regard is how the enactments SVP can promote social, emotional, and meaningful learning experiences in PE.

While academic learning and high stakes assessment commonly dictate and influence approaches to teaching and learning in mainstream and alternative education settings (Berry, 2011; Flower et al., 2011), SVP promoting social and emotional learning (SEL) can help students “learn and apply a set of social, emotional, behavioral, and character skills required to succeed in schooling, the workplace, relationships, and citizenship” (Jones et al. 2017, p. 12). Increased focus in PE has been given to how SEL theory and practices are explicitly understood and implemented by teachers with students (Wright and Richards, 2021; Wright et al., 2021a,b). Simultaneously, there is increased advocacy for pedagogical approaches “positioning the personal, affective, and intrinsic meanings of learners at the core of curriculum development and pedagogical enactment” (Ní Chróinín et al. 2018, p. 119). Student voice pedagogies can help engage and affect students to become more motivated and learn about physical activity, movement, and well-being in an invested and embodied manner (Long and Carless, 2010). Yet, despite the rhetoric, PE has “yet to maximize its potential with regard to the development of SEL competencies” (Hooper et al. 2020, p. 140). Similarly, research also indicates that there is a lack of understanding as to how meaningful physical education (MPE) can be promoted and accomplished (Lynch and Sargent, 2020), with a lack of contextually relevant empirical data drawn from the individual experiences of children and adolescents (Fletcher et al., 2021; Ní Chróinín et al., 2021). Drawing on social constructivist learning theory in understanding and implementing a MPE approach, and a systemic framework for SEL, the purpose of this study was to explore learners' experiences enacting SVP (i.e., full value contract, personal biographies, cooperative learning and group processing, continuous class consultation and negotiation, timelines, taster sessions, photovoice, written, and digital reflections) to promote SEL and MPE in an alternative education setting. Two research questions guided the research process: (1) How did students interpret and enact these pedagogies? (2) What contribution did the enactment of these pedagogies have in promoting SEL and MPE?

## Theoretical Framework

In order to consider how enacting SVP promotes MPE among students, we draw on social constructivist learning theory as “an appropriate theoretical basis upon which to ground its teaching and learning principles” (Fletcher et al. 2021, p. 6). Students' personal experiences are often framed within a socially interactive PE environment and the social support received from both peers, teachers, and others inside and outside of the class which can enhance meaningful engagement with content (Gibbons and Gaul, 2004; Beni et al., 2017). Doing so requires us to “understand the multiple cultures of the learner, teacher, school, and society; how these impact learners; and how to plan curriculum and instruction that leads to robust, meaningful knowledge useful in multiple contexts” (Rovegno 2006, p. 271). Such a view posits that students can become active, dynamic, and democratic agents within the classroom, adapting, and developing practices and interactions to understand and experience SEL and MPE in PE (Kirk and Macdonald, 2009; Azzarito, 2016). Drawing on the features of MPE recently articulated by Beni et al. (2017) and stemming from the work of Kretchmar (2000, 2007) and Metheny (1968), a MPE approach rests on the deliberate prioritization and inclusion of five features when designing and implementing teaching and learning: social interaction, fun, challenge, motor competence, and personally relevant learning. Such an approach requires practitioners and researchers to align pedagogy with the affective domain through allowing “individuals ascribe meaningfulness by making sense of past, present, and future experiences (including interactions with self and others, artifacts, content, and pedagogies) through a process of synthesis and reconciliation” and an emphasis on “the individual and the contextually-bound nature of a meaningful experience” (Beni et al. 2017, p. 292); requiring “a focus on meaningful experiences and the process of making new or revised meanings out of experience” (Quennerstedt 2019, p. 619). In this regard, Ní Chróinín et al. (2018) have encouraged researchers to further explore “the value of making the prioritization of meaningful experience explicit through modeling and discussion, engaging with meaningful experiences as both a teacher and learner as well as reflecting on those experiences” (Ní Chróinín et al. 2018, p. 131). Central to MPE is the deliberate enactment of democratic and reflective pedagogies which embody SVP approaches and help students engage and interact with others in an enjoyable physical activity environment (Ennis, 2017; Fletcher and Ní Chróinín, 2021; Ní Chróinín et al., 2021).

**Figure 1:**
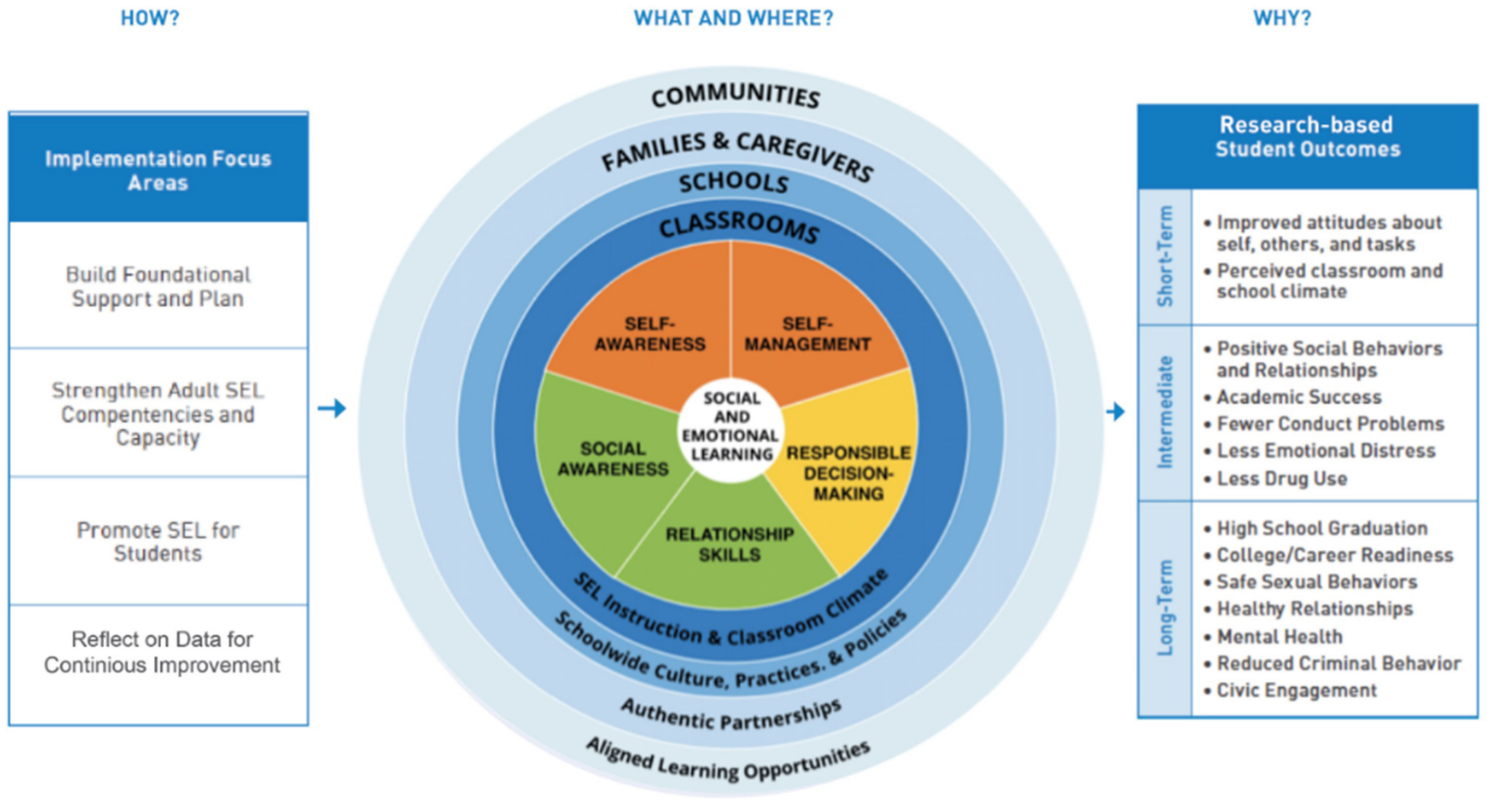
CASEL Framework for Systemic SEL (Collaborative for Academic, Social and Emotional Learning, 2015).

For SEL specifically, we draw on *The Collaborative for Academic, Social, and Emotional Learning's (CASEL) Framework for Systemic Social and Emotional Learning* (Collaborative for Academic, Social and Emotional Learning, 2015; Borowski, 2019; see Figure 1). The CASEL Framework presents “comprehensive multi-dimensional framework of the skills essential for successful social and emotional development” and a foundation for guiding the implementation of evidence based SEL pedagogies (Ross and Tolan 2018, p. 1188). Bridging educational theory with practice, the framework identifies five interrelated sets of cognitive, affective, and behavioral competencies (self-management, self-awareness, social awareness, relationship skills, and responsible decision making) (Weissberg and Cascarino, 2013; Dusenbury and Weissberg, 2017; Blyth et al., 2019). These competencies involve the targeting of specific skills which, when logically blended into teaching and learning, can help facilitate the holistic accomplishment of broader learning outcomes beyond physical and cognitive subject matter. Within the school and classroom context, the teacher's pedagogical skills are crucial in accomplishing child level outcomes, leading to potentially improved child-level impacts. However, challenges in doing so are widely acknowledged, with the successful accomplishment of SEL dependent on factors such as time, cultural and contextual sensitivity, and designing and implementing effective pedagogies with practitioners and students (Blyth et al., 2019; Kaynak, 2020). We posited that if all of these above premises held true, then deliberately and consistently enacting SVP and reflecting on them with students could help provide us and them with some valuable insight through which to understand and enhance SEL and MPE experiences both in the present and going forward (Rovegno and Dolly, 2006; Ennis, 2017). In the next section, we present the methodological approach which guided this study.

## Methodology

### Research Design

This study implemented a qualitative case study design (Stake, 2013) framed by a participatory action research (PAR) approach spanning 12 weeks from February to May 2021. The convenience sampling procedure was used for ease in accessibility to participants (Cooksey and McDonald, 2019). It was conducted as part of a larger study exploring teachers and students' understandings and experiences of SEL and MPE. Participatory action research is “a research design and philosophy that seeks to produce knowledge and action with participants and use this knowledge to improve the life circumstances of research participants during the course of the research itself” (Enright and O'Sullivan 2012a, p. 129). We view PAR as an essential component of any research design that claims to enact SVP. In the oncoming sections you will read how the enactment of SVP and the research methods utilized for data collection went hand in hand with each other during the research process. Doing so ensured participants had opportunities to be included and participate in identifying, addressing, and responding to pre-existing, emerging, consensual, and contested teaching and learning experiences which arose before, during, and after the PE classes and research process (Enright and O'Sullivan, 2010b; Fitzgerald et al., 2021).

### Setting and Participants

In line with the ethical procedures approved by the school district and University's office of research and integrity, both students and the school have been assigned pseudonyms. The research was conducted in Tyber College an urban alternative high school with 250 students, operated in partnership by the local school district and a University where the school's campus was situated. Within alternative education settings, there tends to a higher prevalence of health-risk behaviors, social and emotional problems, and a paucity of research focusing on behavioral interventions (Olsen, 2010; Johnson and Taliaferro, 2012; Schwab et al., 2016). The U.S. Department of Education defines alternative education schools as any “public elementary/secondary school that (a) addresses the needs of students who typically cannot be met in a regular school; (b) provides flexible/hybrid education opportunities; (c) serves as an adjunct to a regular school; or (d) falls outside the categories of regular education, special education, or career/technical education” (National Center for Education Statistics., 2016). Participants in this study included 16 ninth grade alternative high school students (eight girls/eight boys) aged 14–15 from a class of 18 who had just returned to face-to-face learning in January 2021 for the first time following COVID-19 restrictions and closures in March 2020. Tyber typically enrolls underserved/at-risk students from the local school district and through long established University partnered youth development programs. The USA Department of Education (United States Department of Education^1^) defines this population as:

Students at risk of educational failure or otherwise in need of special assistance and support, such as students who are living in poverty, who attend high-minority schools, who are far below grade level, who have left school before receiving a regular high school diploma, who are at risk of not graduating with a diploma on time, who are homeless, who are in foster care, who have been incarcerated, who have disabilities, or who are English learners.

Further details on each student, their gender, race, ethnicities are presented in Table 1. This detail is drawn from the personal biographies they submitted and shared with each other at the beginning of the course in class. In doing so, we emphasize the need for researchers to ensure participants' voices and input is included across all aspects of student voice research processes—not just the findings.

**Table 1:** Participant details.

**Student**	**Gender**	**Race/Ethnicity**
Barry	Male	Caucasian, Christian
Sarah	Female	Caucasian
Jess	Female	African American, Native American
AJ	Male	Hispanic
Aamira	Female	African American, Muslim
Jack	Male	African American
Khalid	Male	African American
Cora	Female	Caucasian, African American
James	Male	Caucasian
Channing	Male	African American
Auria	Female	Caucasian
Alisha	Female	Hispanic
Melissa	Female	African American
Aubrey	Female	African American
Leo	Male	African American
Landon	Male	African American

### The PE Course and Pedagogies

The outline of the PE course was designed by a group of five researchers and practitioners from the University. Collectively, the group of five had combined practical experience of teaching PE across multiple international K-12 and higher education settings, ranging in experience from 4 to 20 years. This comprised of the first author and second author, the third author/designated PE teacher, the fourth author and teacher education professor, and the fifth author. It was purposefully designed with the intention of developing students' understanding and application of SEL and MPE and physical activity. Central to this was the enactment of SVP. Similar to the recent work of Lynch and Sargent (Lynch and Sargent, 2020; Sargent and Lynch, 2021) in higher education UK settings, a number of what the research team regarded as SVP were drawn upon from their own collective experiences of enacting student voice and teaching SEL and MPE in the field. These pedagogies were modified in order to be made more appropriate and then collectively implemented frequently, or specifically at different points throughout the course. These are presented in Table 2 and further detailed in a Supplementary Material. For example, Cooperative Learning (Dyson and Casey, 2012, 2016) was used as the primary pedagogical method during weeks 1–4 when the focus was on building relationship skills with students in class. Structures implemented included *Learning Teams, Jig-Saw, Think-Pair-Perform*, and *Rally Round Robin*. Particular attention was paid to implementing the cooperative element group processing throughout the course, typically in the form of “an open dialogue or group discussion related to the lesson content that can occur at any time during the lesson” (Dyson and Casey 2012, p. 4). These structures were frequently used throughout the remainder of the course at different times, but alongside other pedagogical practices such as peer tutors, task stations, direct instruction, intra-task variation, mastery learning, play-teach-play, and child designed activities (Graham, 2008). The course itself was implemented across 10 weeks February to April in sixteen 75 min lessons, typically delivered twice a week depending on the school calendar. The enacted pedagogies were implemented in class as well as asynchronously using the school's online CANVAS platform.

**Table 2:** Enacted pedagogies drawn from literature and research prioritizing student voice.

**Week(s)**	**Pedagogy drawn/modified from literature**
1–2	Full value contract (Tannehill and Dillon, 2009)
1–2	Personal biography (Betourne and Richards, 2015; Sutherland and Parker, 2020)
1–10	Cooperative learning and group processing (Dyson and Casey, 2012, 2016; Sutherland et al., 2019)
2–10	Continuous class consultation and negotiation (Enright and O'Sullivan, 2010a,b; Howley and Tannehill, 2014; Howley and O'Sullivan, 2020, 2021; Aarskog et al., 2021)
3–4	Timeline (Enright and O'Sullivan, 2012b)
4–7	Taster sessions (Enright and O'Sullivan, 2010a,b; Howley and Tannehill, 2014)
7–10	Photovoice task 1 (Enright and O'Sullivan, 2012b; Azzarito and Kirk, 2013)
9–10	Photovoice task 2 (Enright and O'Sullivan, 2012b; Azzarito and Kirk, 2013)
2–10	Digital reflections (Lynch and Sargent, 2020; Sargent and Lynch, 2021)
10	Overall digital reflection (Lynch and Sargent, 2020; Sargent and Lynch, 2021)

### Data Collection and Analysis

In line with a PAR approach, this study utilized a range of traditional and innovative participatory qualitative research methods including students' personal biographies, timelines, digital and written reflections, photovoice, and class artifacts which had been completed as part of course work. In implementing these methods and in line with the purpose of this study, we aimed to “go beyond simply conversing with young people” about what their experiences in PE look like (Enright and O'Sullivan 2012a, p. 122). After the implemented course and grading process was complete, the first, second, and third author/designated PE teacher contacted students and presented information on the larger research study as part of a retrospective recruitment process which had been ethically approved by the local school district and the University's office of research and integrity. Following the completion of parental consent and minor assent, 16 of 18 students in the class agreed to participate in the study. Their personal biographies (Safron and Landi, 2021), timelines (Safron and Landi, 2021), photovoice task 1 (Drury et al., 2017), photovoice task 2 (Safron, 2020), digital reflections (Weissberg and Cascarino, 2013), and overall digital reflections (*n* = 15) were downloaded from the CANVAS platform, transcribed, de-identified, and stored safely. Each student's data set was transcribed and then presented to them in the focus groups which were conducted at the end of the course and engaged students in reflection of their experiences. The Miles, Huberman, and Saldana Framework for Qualitative Data Analysis involving data condensation, data display, and drawing and verifying conclusions was initially implemented, with thematic analysis (Miles et al., 2014; Richards and Hemphill, 2017). This involved both deductive and inductive combination of comparative and thematic analysis, or abduction. Abduction is a process of mixing data-based inductive analysis and theory-driven deductive analysis, which combines the deductive and inductive models of proposition development and theory construction (Astbury and Leeuw, 2010); a “constant shuttling between theory and empirical data, using both inductive and deductive reasoning” (Astbury and Leeuw 2010, p. 374). While drawing on our theoretical and conceptual frameworks to deductively analyze data, we also relied on inductive reasoning to seek out patterns and themes which were generated outside of this.

Before presenting results, we wish to acknowledge that the dependability, credibility, transferability, and confirmability of the data is limited in so far as it relates to only to these students in this particular class who worked directly with the third author and designated teacher, and later first author. Regarding bias, neither the first author or the third author and designated teacher had worked with this group prior to the intervention. The first author did not interact with the participants until seeking consent and conducting the focus group interviews and was primarily responsible for analyzing the accrued data set. The dependability of findings might be influenced by the return of face to face learning and having to implement social distancing with the PE setting which in itself was a new experience for both the teacher and students to contend with. Still regarding dependability, we have drawn from previous studies in the field which have affirmed these pedagogies and participatory research methods both in isolation and in smaller combinations with singular and multiple groups. We have presented information on participants, and how these pedagogies and methods how were consistently implemented, both here in the paper, and in the Supplementary Material. Data from every one of the 16 students who consented to participation in the study out of a total of 18 in the class have been presented in this paper; from a minimum data source of one for both Audrey and AJ, to a maximum of six for Aamira, Alisah, and Jack; making for an average utilization of over three data sources per student to make up the 57 data sources presented next in findings. The multiple methods and range of sources utilized right up until the focus groups to enact each students voice helps to triangulate and confirm these methods. The second author served as peer reviewer and regularly debriefed with the first author and evaluated his rereading and probing of the data. The third author and designated teacher also acted as a peer reviewer in interrogating and confirming the findings. The fourth author served as a critical friend in designing and implementing the course activities and did not interact with the students. The fifth author oversaw the implementation of a soccer taster session. This study represented the research team's most concerted attempt to date in understanding and implementing these combinations of pedagogical and research approaches collectively and in sequence. In addressing all these standards to affirm and justify the quality of our conclusions, we echo Enright and O'Sullivan's (2010a) view that “reliability, validity, and ethical acceptability of research with young people is enhanced by using these types of methods that facilitate students in shaping the research agenda and are deemed by young people as relevant and interesting methods to engage with their realities” (Enright and O'Sullivan 2012a, p. 126). In this way, we emphasize the need to also be transparent and upfront with you, the reader, every bit as much as we were with the students we worked with in the research process when presenting findings from this kind of work, which follows next.

## Findings

The following themes were constructed as thematic findings representing students' experiences of enacting SVP to promote SEL and MPE: *Making responsible decisions, unearthing and sharing mixed emotions, picturing physical activity beyond the classroom, recognizing the role of relationships, considering challenge and competence*, and *pursuing meaning*.

### Making Responsible Decisions

Students recognized their role in making responsible decisions, ranging from establishing rules, routines, and expectations, regular group processing, and making decisions on selecting and negotiating content: “We worked on discussing our values regarding the class” [Auria, Reflection (REF) 1]; “We got to do what we wanted, like to vote and stuff like that” [Khalid, Focus Group (FG)B]; “It was like a main focus to make sure that everyone felt like they had some say” (Barry, FGC). Students cited having the opportunity to critique and modify class content and make caring and constructive choices about personal behavior and social interactions in different situations: “It was pretty student led…but it wasn't just like us by ourselves coming up with our own games. It was us as like a group” (Leo, FGC); “I liked how it was in our hands…there was some things that we realized we could critique” (Jack, FGD); “How we changed the hockey game a little bit, it was a little bit easier for me than to like play an actual game” (Julia, FGD). This was especially facilitated through frequent group processing after tasks: “We used that in our reflection [group processing] when it went wrong and how we fixed it and what we learned from it too. I think that's a good part. Learning from your mistakes” (Leo FGC). Providing students with continuous opportunities to reflect on and discuss their learning and make decisions on their future learning experiences required students like Barry to be responsible and aware of how such decisions affected the group collectively:

“When I play a game that I already know how to play but others don't, I have to help them understand how to play the game properly…I think that in the future I will be more willing to help others learn about a sport that they may not have played before” [Barry, Overall Digital Reflection (ODI)]

Cathy saw the process of facilitating student decision making as beneficial not just for students, but for the teacher also:

“If you have a good understanding of what your students like and what they don't like and what some students can do, and like what all students can do, like you can have fun and people will enjoy it more…it's about listening and understanding people as well” (Cathy, FGB)

In making these decisions students also had to consider theirs and others pre-existing and emerging emotional attachments to PE and physical activity.

### Unearthing and Sharing Mixed Emotions

The implementation of pedagogies evoking individual and group reflection helped students develop a sense of self and social awareness with regard to their collective experiences and relationships with PE and physical activity. Students consistently reflected on and described the array of emotions they and others had experienced: “I started to do tennis…I eventually gave that up too…I had really low self-esteem at that time and I always felt like giving up” [Cathy, Timeline (TL)]; “I do feel that [basketball] helps me develop socially and emotionally…I develop emotionally by accepting a challenge instead of feeling defeated” [Auria, Photovoice Task(PT)1]. Opportunities to reflect on their learning experiences in class allowed students to unpack and understand theirs and others emotional states: “I don't like doing physical stuff in front of people” (Alisha, REF1); “After we were done [meditation] my body felt loose and I felt very calm…I think the others were feeling the same way” (Channing, REF2); “Everyone was kind of frustrated since the game wasn't as fun as we thought” (Aamira, REF7). Students recognized the vital role of reflecting on and understanding these experiences: “It made you really think if your PE experiences in the past were good or not…if the person had a bad experience” (James, FGD); “Different emotions started to come into play…I just started to think back about different things that happened…yeah, it was like a wow moment” (Landon, FGD); “The environment that she had set, that it made it easy to interact and have emotion” (Jack, FGD); “When I do something, like, I always want it to like affect me in some type of ways, like have like some type of meaning” (Channing FGA). Reflective tasks allowed students to consider the connection and influence of emotions in past experiences and how these both positively and/or negatively influenced and informed their present movement experiences. Through considering and sharing the range of emotional experiences they had previously encountered when engaging in PE and physical activity, students were able to develop a deeper sense of self and social awareness with regard to how their emotions influenced their movement experiences. This was especially encapsulated by Alisha in her ODI, where she describes learning to self-regulate her emotions over time and develop a growth mindset around her participation in movement experiences.

“I used to have a bad mindset about myself. I wouldn't even want to play with my friends because I was nervous my own friends would judge me. I've grown from that. I now enjoy and do things worry free…it was a bad feeling but now that I've outgrown these emotions and thoughts I look back and think about how I felt and it makes me not think badly about myself…I've learned many different movements in PA that I've used in my personal life” (Alisha, ODR).

Understanding the emotions students and how this affected their relationship experienced inside and outside of PE in turn asked students to consider their relationship with physical activity outside of the classroom.

### Picturing Physical Activity Beyond the Classroom

**Figure 2:**
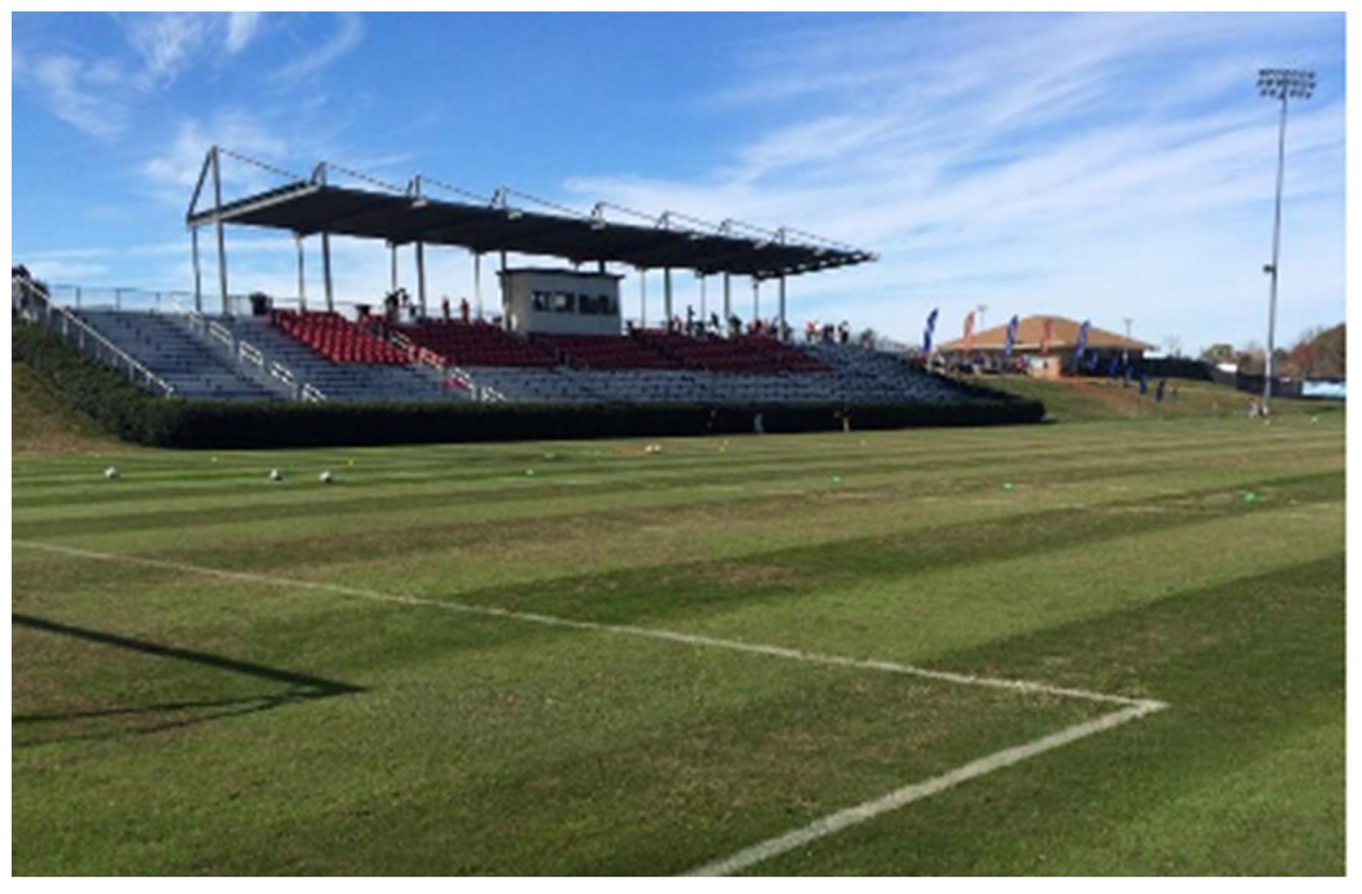
“I'm a soccer player and I play on a high level academy team. I play every day and I have games on the weekend in the stadium that is in the picture. This is where we play our home games. This is my physically active life.” (Jack PT1).

**Figure 3:**
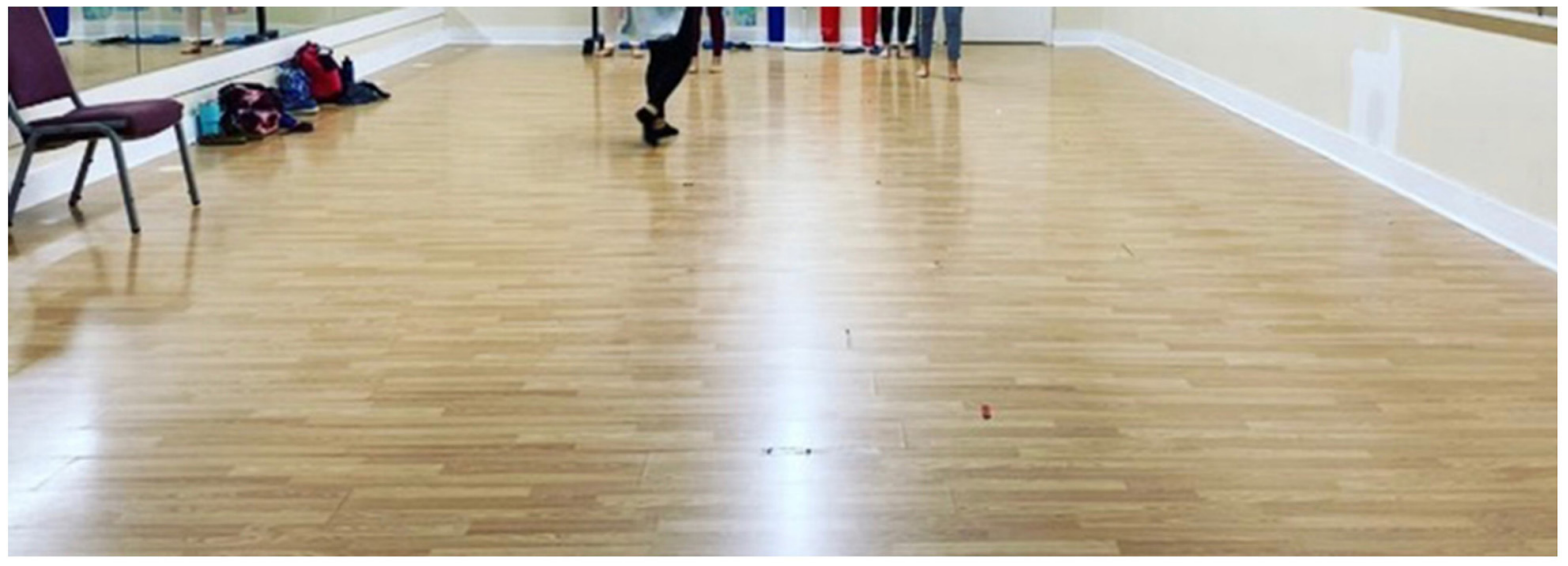
“This is the floor I do both pointe and ballet on as well as modern. It's a wooden surface for the use of nice slides and movements. With socks on the floor can be slippery like all wooden floors are with socks but overall, this is my favorite type of floor to do turns and slides on.” (Sarah, PT2).

**Figure 4:**
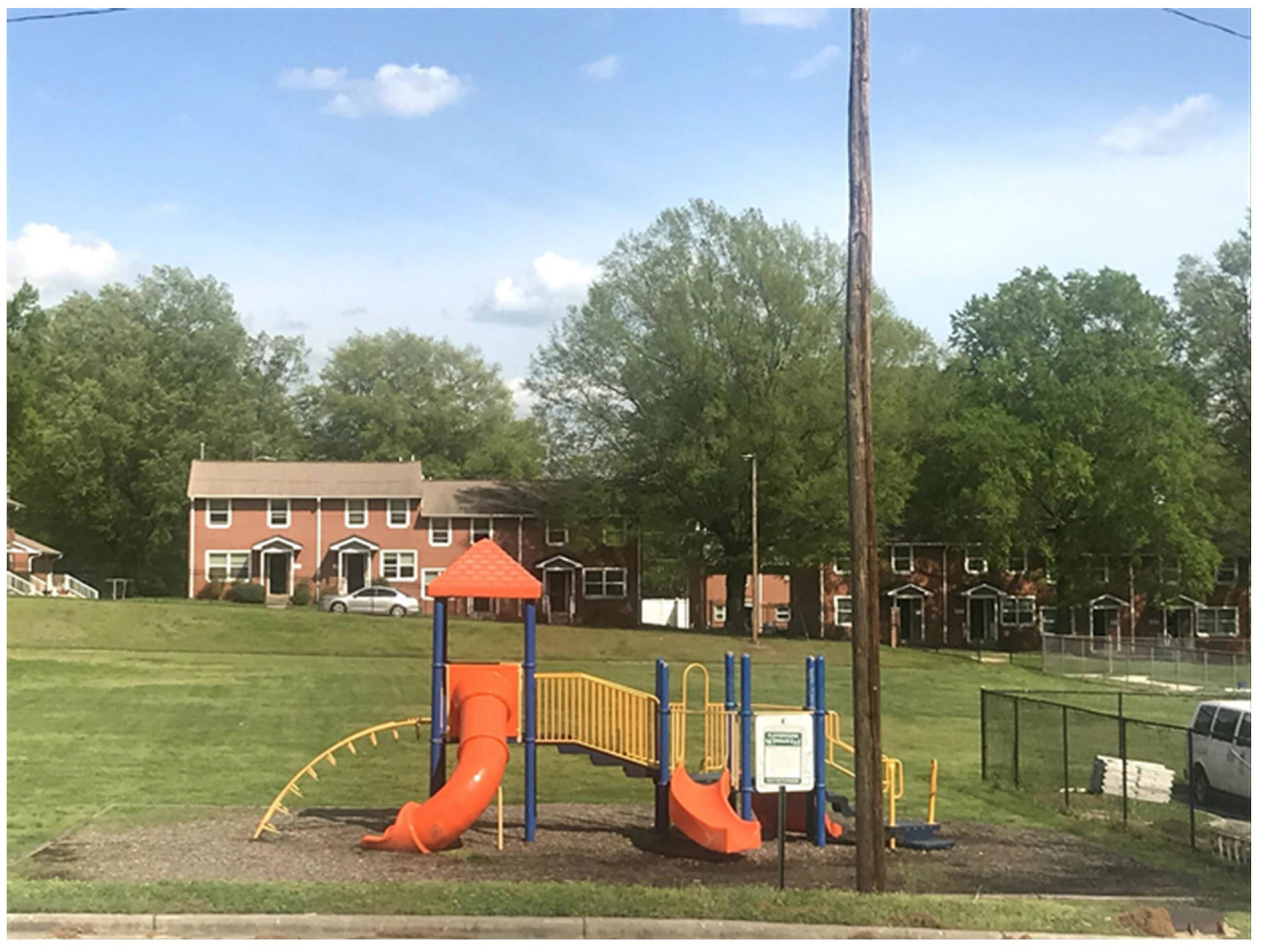
“I don't really have much of a physically active life I'm more of a reader. I do try to go on walks every day and I take my little sister and nephew to the park to play around. I still have to chase after them a lot sometimes so I guess that's me being physically active.” (Aubrey, PT1).

**Figure 5:**
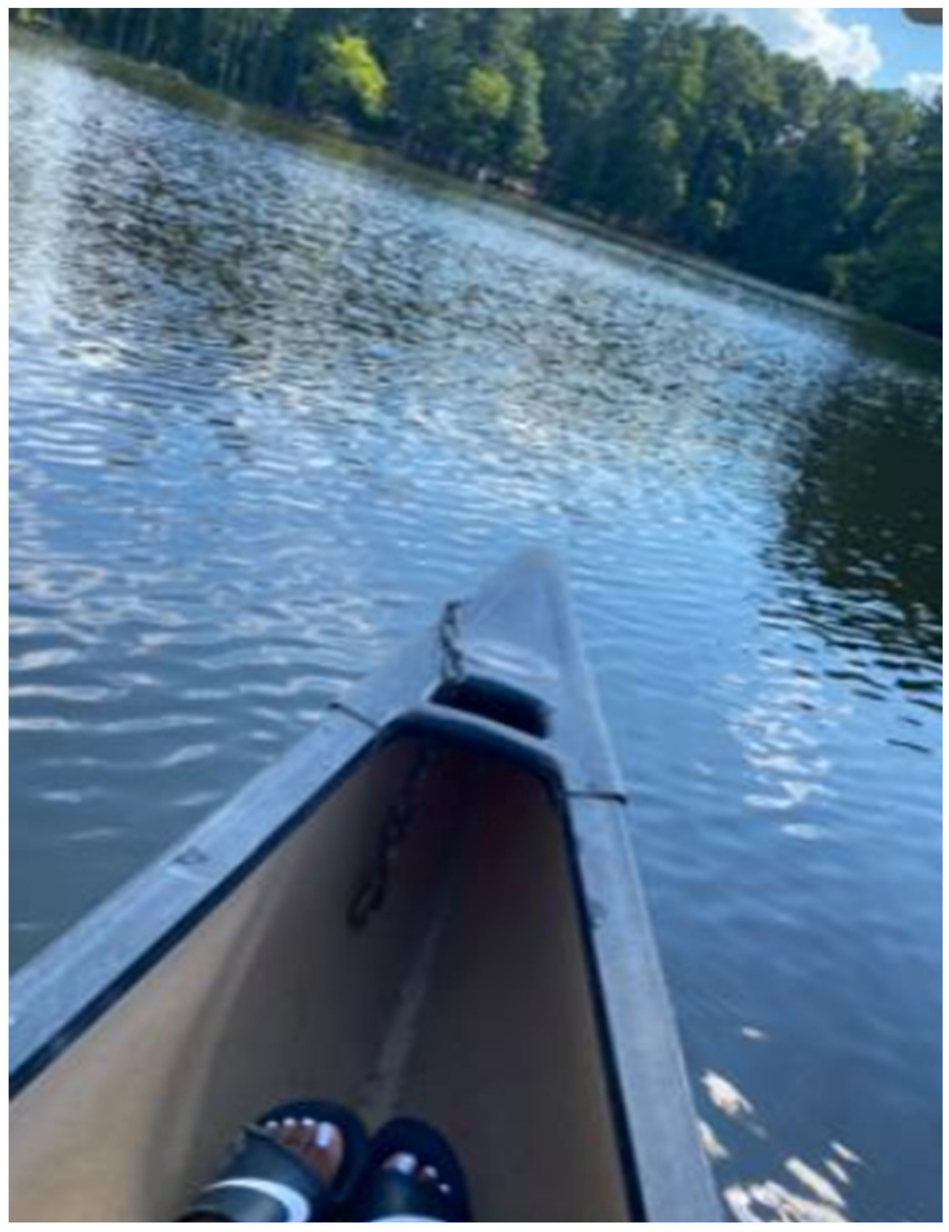
“Physical activity facilities nearby: One of the physical activity facilities near me is a park where you can canoe and kayak. This is really important to me because my family loves doing outside activities.” (Julia, PT1).

**Figure 6:**
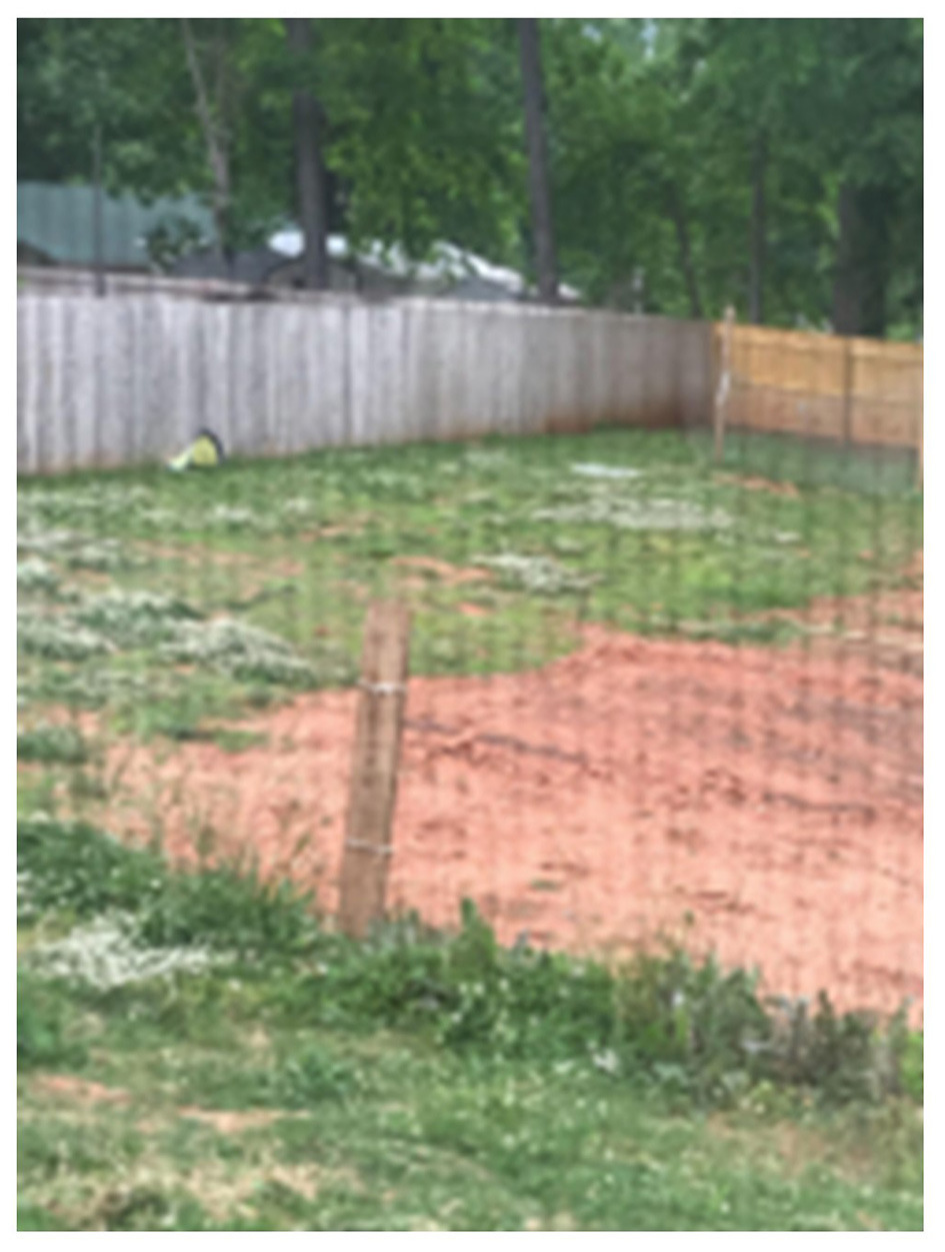
“I play soccer outside in my backyard with my two brothers” (Khalid, PT1).

**Figure 7:**
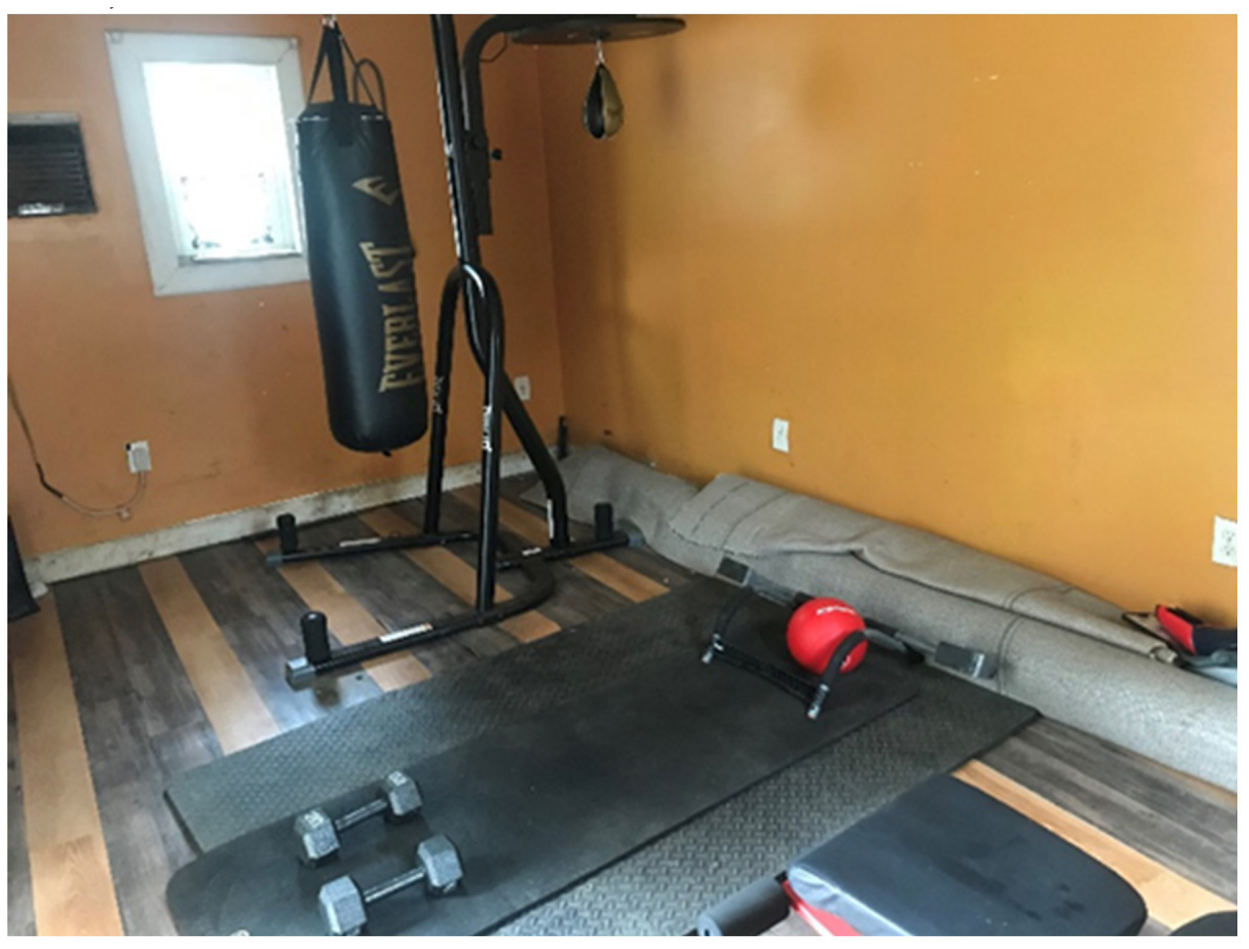
“This ties in with my physical activity life because this is where I work out three times a week for an hour” (AJ, PT1).

**Figure 8:**
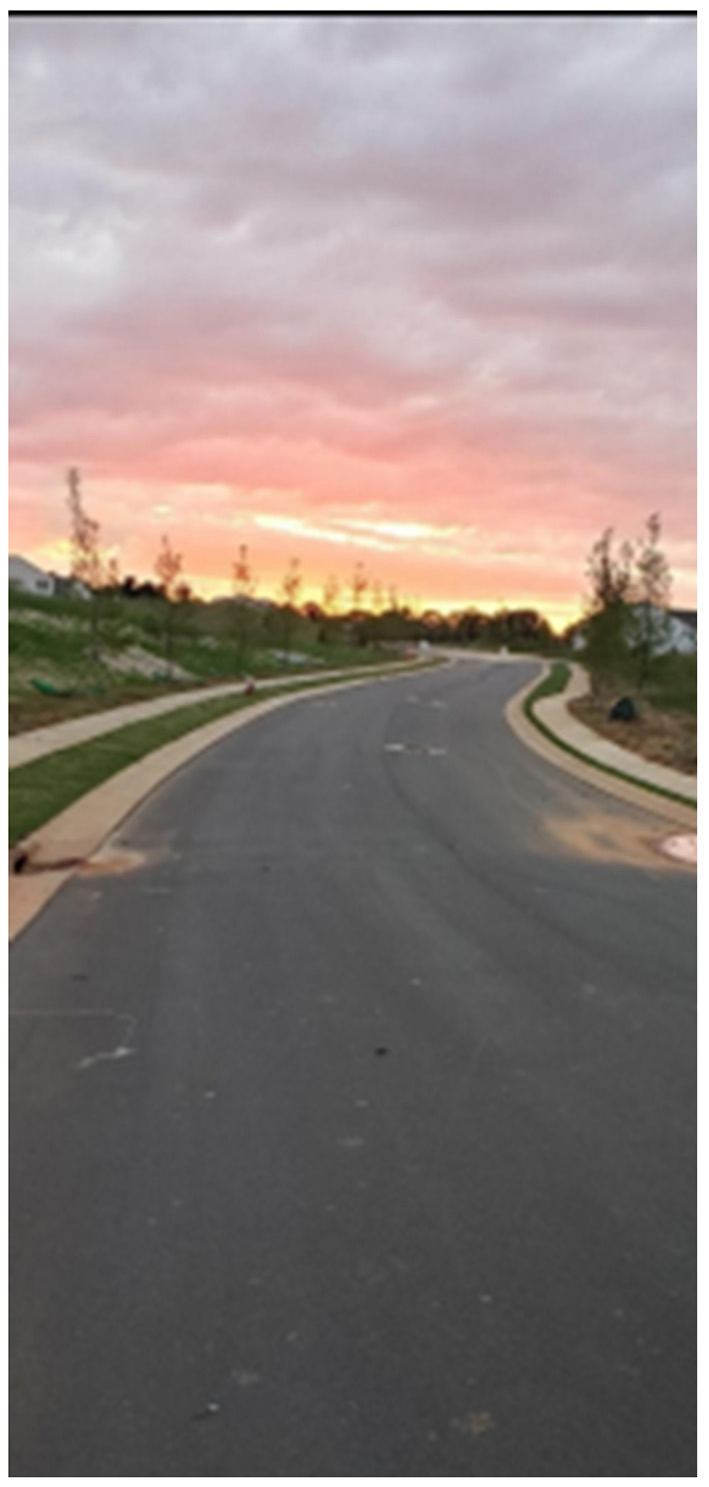
“My physical active life: It's quite literally not there besides walking around in my neighborhood some, that's about it. This class has made me realize I really need to start working out more!!” (Cathy, PT1).

**Figure 9:**
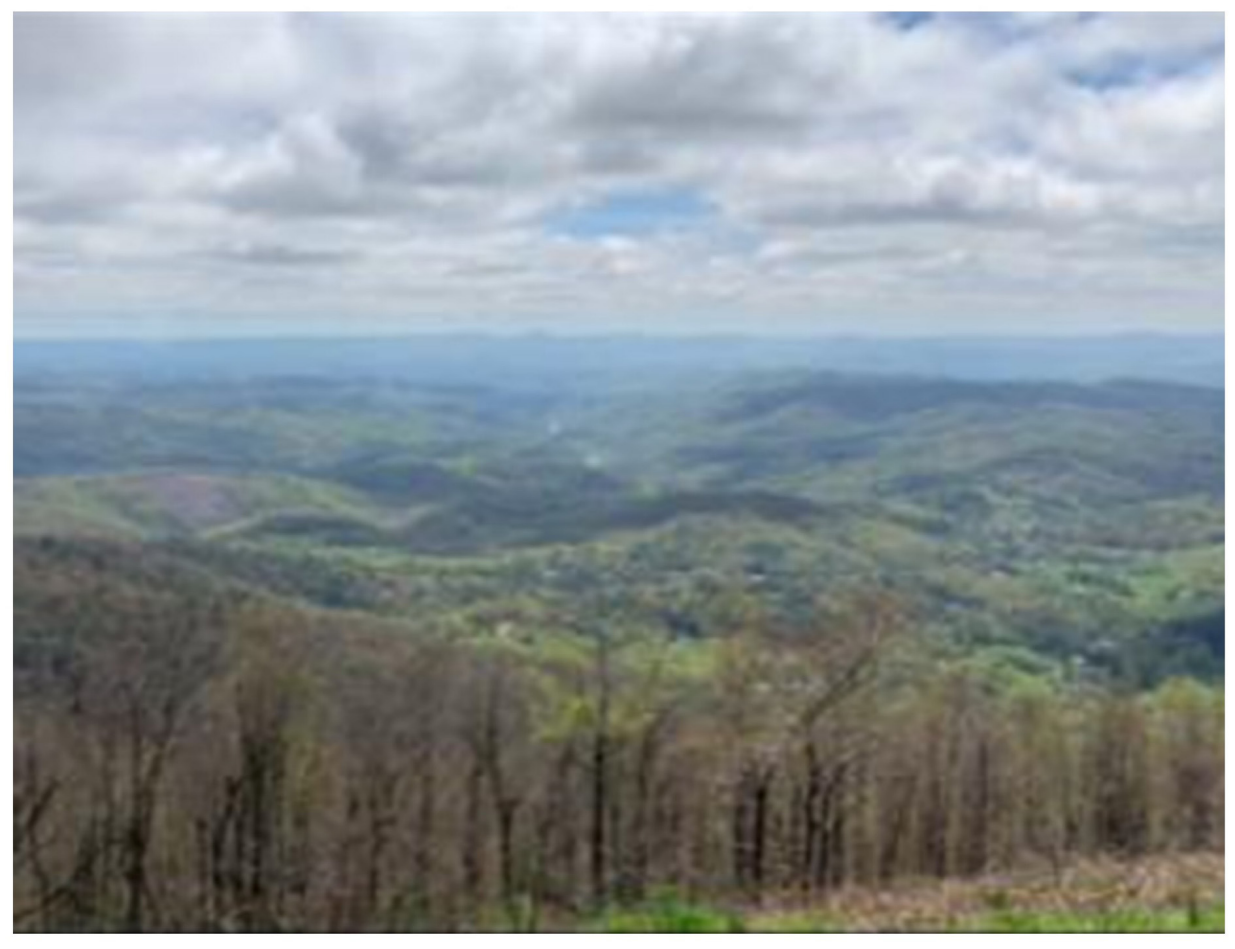
“Physical activity in the lives of my family and friends: Last summer, my family and I hiked a mountain and once we reached the top, we took this picture. Doing physical activities with my family is fun and also helps me stay active.” (Julia, PT1).

Facilitating students in articulating and illustrating how they pursued physical activity and movement in their lives outside of PE promoted self and social awareness and demonstrated their unique and colorful experiences, many of which varied greatly from those which they encountered in PE: “Outside of school, you kind of go your own way since you don't really have like a coach or a gym teacher” (Aamira, FGC). This allowed students to better understand and establish how they maintained active and healthy lives outside of the PE course through identifying additional significant movement experiences. Students such as Jack and Sarah pursued physical activity through formal participation in soccer and dance (see Figures 2, 3), while students like Aamira, Aubrey, and Julia utilized local recreational facilities informally: “I mostly just go to the park, sometimes I bring my ball and play in the field, most of the time I just swing or do the monkey bars” (Aamira, PT1); Aubrey (see Figure 4); Julia (see Figure 5). For others like Khalid and AJ, they utilized their homes and local environments (see Figures 6, 7). This helped students make sense of their physically active lives beyond PE. Notably, the photovoice tasks helped students like Cathy (Figure 8) and Julia (Figure 9) recognize the nuanced ways in which they were physically active whereas previously they didn't see themselves as being so:

“During the photo voice activities, I was able to learn that like I was doing more physical activities during my lifespan than I actually thought I was; I was going to the gym more often and like me and my family, we would go and hike or we would go and like walks on trails. I just I didn't think about stuff like that” (Julia FGD).

Through deeper exploration of their worlds, students were able to better understand the role physical activity played in their lives and the lives of those around them.

### Recognizing the Role of Relationships

Students repeatedly reflected on and expressed the importance of social interaction and relationship skills, consistently alluding to positive experiences they experienced in class which enhanced learning: “I felt comfortable with my teammates, and they brought a smile to my face and many laughs…I can be myself around other students” (Sarah, REF1); I learned that with group activities and working together we can actually accomplish many things. I like working with groups I think it's really great.” (Aamira, REF1); “I learned that the more your peers get opportunities to practice, the better they get” (Jack REF4). Students emphasized the importance of supportive relationships and sensitively navigating learning experiences with peers: “Being able to laugh, smile, even struggle with someone can make everything so much better” (Alisha, ODR); “You just had to know when to back off and then know when to push forward and, yeah, let others shine” (Melissa, FGA); “Learning to interact with people on different levels, coming from different backgrounds…learning to be a patient, to help others even if they're on higher or lower level” (James FGD). This was especially the case when dealing with moments of frustration, which occurred throughout the course. An example of this was toward the end of the course when students were asked to create their own games and share them with the class: “We played our game, it was interesting, everyone was kind of frustrated since the game wasn't as fun as we thought, but it was ok” (Aamira, REF7); “The emotions that I was going through triggered plenty of misunderstanding at the start while people were explaining their game. But, after I understood how to play them, I started to have fun playing” Khalid (REF7). The need to practice teamwork and collaborative problem-solving, resolve conflicts constructively, and offer support and help when needed was a regularly observed by alluded to by students, encapsulated here by Jack in his ODI:

“If you don't work together then it's not fun and it gets frustrating…when we played volleyball, you needed to communicate to see who was going to get the ball. If you didn't then your team wouldn't be successful…you have to work harder to help your team when this happens”

Reflective tasks also helped students identify and share how friends and family influenced their movement experiences: “I started working out with my brother and his girlfriend…it was fun working with him; it made me realize working with someone you know can make it more fun” (Alisha, TL); “My brother is in a soccer team, and when he's home, my sister plays against him, and I would be the goalkeeper…we're basically a soccer family” (Aamira, PT 1); (Julia, see Figure 9). The need for social interaction and relationship skills was especially useful when experiencing moments of challenge which affected their levels of competence.

### Considering Challenge and Competence

The SVP implemented allowed students to reflect on and consider the variety and level of challenge they faced in performing different movements and tasks: “Some challenges I faced were trying to remember people's names” (Jack, Ref2); “[Soccer] is a challenge for me because I don't really have any type of foot and eye coordination” (Melissa, PT2); “The challenges I faced this week was struggling to hold the hockey stick the correct way at first, but I was later able to do it” (Auria, REF4); “I've learned that physical education and physical activity is challenging when I don't understand the thing we're doing, it makes it difficult to have fun and enjoy the game if I'm stressed out” (Cathy, ODR). Overlapping with this, was the opportunity for students to also appraise their levels of competence in skill performance when challenged. Within classes, students regularly reflected on challenges they faced in their movement experiences and how this influenced motor competence: “When the class played basketball that tested my motor competence to improve because I was working on passing and dribbling better and learning new techniques” (Khalid, ODR). Sarah considered the role of challenge and cognitive and motor competence outside of class in performance of ballet:

“Ballet is a challenge because you're constantly thinking while doing other things; you think about arm my arms in the right position? What are my facial expressions? Are my toes pointed? What's the next move? Am I spotting? Am I doing this correct? What is my body posture? All these thoughts are going through your head” (Sarah PT 2).

Facilitating students to continuously appraise their experiences of challenge and motor competence prompted students like Landon to think more deeply about how they experienced meaningful movement in class and physical activity:

I'd never knew what motor competence was until I went to this school. I've never ever talked about it in any PE class. So it's like, we're starting to learn these new things and starting to apply it to what we're doing” (Landon, FGA).

In considering challenge and motor competence, students were able to better articulate their movement experiences, moving toward a better understanding of how PE and physical activity was meaningful for them.

### Pursuing Meaning

By the end of the course, students were able to develop and articulate a deeper understanding of what made PE and physical activity meaningful for them; “I've learned that physical activity is more meaningful than I thought it was. It can help you gain social skills, leadership skills, and obviously have positive effects on your health” (Alisha, ODR); “It needs to focus on mental health more instead of physical ability… make it a way to say: ‘It's okay if you can't do it'; like make everyone feel comfortable with their body and their capability” (Auria, FGB). Facilitating students to reflect on, elicit, and share their previous and current experiences while also looking forward helped them consider how to continue shaping and influencing their pursuit of MPE and physical activity: “I never really had to like reflect on what I've done with my physical activity. So it felt different in a way” (Channing, FGA); “It started making me think about the future and stuff like that—how PE is going to affect my life” (Landon, FGA); “It makes you reflect on your experiences with PE in the past. And like, maybe how that kind of could have affected you today and how you use PE that you've learned previously and apply that now” (Cathy, FGB); “I feel like it ties in with what we're doing in the future…I can look back and say ‘Oh, my high school PE teacher taught me this…introduced me to new things that I never thought I would try” (Melissa, FGA). For Alisha, taking what she had learned in PE and transferring it to her life outside the class made for personally relevant and meaningful learning and experiences:

“When I take the knowledge that I've learned from PE and I apply it outside of school I know that's when I've taken the class to a personal level…. throughout my school years I've made memories and experiences, movements, motions in PE that I can look back on.” (Alisha, ODR).

Ultimately, the enactment of SVP allowed students to consider how PE was meaningful for them and the role SEL played in doing so. We now look to understand and discuss what can be learned from these students' experiences enacting SVP to promote SEL and MPE.

## Discussion

The discussion is organized around the two research questions presented in the introduction of the study.

### How Did Students Interpret and Enact These Pedagogies?

Interpretations of what learning is meaningful lies in what is “constructed and understood by the individual; not in an individual bubble detached from reality but influenced by affective and social–cultural dimensions” (Beni et al. 2017, p. 292). In interpreting and enacting these pedagogies (i.e., full value contract, personal biographies, cooperative learning and group processing, continuous class consultation and negotiation, timelines, taster sessions, photovoice, written, and digital reflections), students came to appreciate and make greater sense of their experiences of PE and physical activity alongside those of their peers. The deliberate and consistent enactment of individual and group discussion and reflection helped ascertain students' prior knowledge and experience to assist in their ongoing learning across multiple domains (Rovegno and Dolly, 2006). As Cathy noted, such a process was not only beneficial for students, but for the teacher also when it came to curricular planning and decision making. Notably, when doing so, students openly reflected on and expressed the array of mixed emotions they experienced and observed when participating in PE and physical activity as individuals, as well as when interacting with others. In interpreting and responding to the intentionally social and affective elements of these pedagogies, their subsequent and consistent enactment helped students elicit and reflect on their current and pre-existing learning experiences more deeply. This assisted them in identifying opportunities and constraints which, respectively, promoted and detracted from their pursuits of physically active and healthy lifestyles. This was a new and novel experience, and something which they had not been involved in during PE previously. Student voice pedagogies successfully elicited and illustrated how students performed movement and physical activity beyond the content of the course itself, helping them to connect what they were doing in PE to experiences across varying social and environmental contexts (O'Connor, 2019). For students like Julia, pedagogies such as the photovoice tasks led students to realize how physically active they were, allowing them to reconsider what being physically active meant, looked, and felt like beyond on their understandings and experiences of PE. This was a new departure, and highlights the need to also directly engage students in what O'Connor and Jess (2020) describe as *border-crossing*; a broad concept of sharing thought, practice and resources within intellectual communities and between contexts (O'Connor and Jess 2020, p. 410). As demonstrated here, embedding SVP in PE has the potential to lend itself to “a broadening of the skills, knowledge and understanding encompassed within curricula and for a lifelong curriculum to be acknowledged as the collective responsibility of organizations and individuals within and beyond existing formal education structures” (Penney and Jess 2004, p. 269). The utilization and selection of a variety of movement activities through the taster sessions, and elicitation, illustration, and subsequent sharing of students' movement experiences outside of class provided students with experiences and opportunities to consider PE and physical activity beyond traditional PE-as-sports techniques/multi-activity PE and “a one-size-fits-all approach” (Kirk, 2013, p. 978).

There is a tendency in PE programs to steer clear of recognizing, understanding, and addressing the range of emotions which manifest themselves within learning experiences (Bailey et al., 2009; Dyson, 2014; Dyson et al., 2020; Hooper et al., 2020). Students came to interpret and understand these pedagogies, which prioritized SEL and MPE, as a necessary part of their learning experience, assisting them in making more inclusive and considerate decisions about class content, physical activity, and how they interacted with others and participated in physical activity. In this way, the deliberate enactment of democratic and reflective pedagogies deepened students' understandings of their experiences and helped them make multiple connections supporting transfer to other contexts in their lives (Rovegno, 2006; Fletcher and Ní Chróinín, 2021). While early days, enacting SVP helped students become successful, active, dynamic, and democratic agents within their learning community, adapting, and developing practices to promote participation in PE and physical activity through these continuous interactions (Azzarito, 2016). Enacting student voice cannot be perceived and implemented as a fixed process but rather a fluid continuum of practice that involves trial and error—an idea largely detached from students' previous experiences of schooling and the notion of fidelity in evidence-based research (Howley and O'Sullivan, 2021). In helping students to engage in, reflect on, and embrace their array of experiences with physical activity and movement, we see how these democratic and reflective pedagogies ultimately led students to slowly but surely identify, reduce, and eliminate “mis-educative or non-educative aspects that detract from participation” within PE (Ní Chróinín et al. 2021, p. 12). While this was by no means a transformative or finished process, students' initial interpretations and enactment of these pedagogies led them to be more engaged and invested in their PE classes and think more deeply about how they participated in PE and physical activity inside and outside of school, encapsulated for example by Alisha's ODR when she considers what she had learned in PE and how it was personally relevant and applicable to her broader life.

### What Contribution Did the Enactment of These Pedagogies Have in Promoting SEL and MPE?

The extent to which SVP are enacted in research by participants to directly improve learning and assessment in relation to PE curriculum often results in democratic practices eclipsed by circumspect curricular practices designed to navigate high stakes examinations (Howley and O'Sullivan, 2020, 2021; Hooper and Sandford, 2021; Iannucci and Parker, 2021). The findings of this study are significant in this regard as they demonstrate a clear connection between the aim of the PE course and the learning outcomes which subsequently transpired through the enactment of SVP. The explicit, deliberate, and consistent emphasis on SEL and MPE when enacting SVP allowed students to develop more uniform understandings and a common language around both concepts and how they related and contributed to PE, physical activity, and students' broader lives. We see from the words of the students how the prioritization of these competencies and features contributed to their broader PE experiences and learning within the subject. Through facilitating students in identifying and sharing previous and present understandings and experiences of SEL and MPE in PE and physical activity participants developed a deeper sense of self and social awareness through identifying and understanding their own emotions, thoughts, and values and how they have influenced their physical activity experiences and behaviors across contexts, while also learning to understand the perspectives of and empathize with others (Collaborative for Academic, Social and Emotional Learning, 2015; Borowski, 2019). This in turn assisted students to make responsible decisions around selecting content and their behaviors when working with others in class to create an emotionally safe and inclusive environment. It also helped them to consider how they experienced and applied SEL and meaningfulness to physical activity outside of class individually, and amongst friends, family, communities, and other organizations. In particular, students acknowledged how the explicit focus on developing relationship skills was crucial to the quality of their learning experiences in PE (Glasby and Macdonald, 2004; Howley and Tannehill, 2014). In prioritizing SVP, SEL became part and parcel of each lesson.

Looking at PE more specifically, encouraging students to reflect on their experiences through a MPE lens allowed them to assess and further understand the significant role each feature had in promoting quality physical activity and movement experiences. Attempting to articulate students' sense of meaningfulness required them consider the “complex mix of individual cognitive and affective elements as well as relational, social, and cultural dimensions” (Fletcher et al. 2021, p. 4). The variety of oral, visual, and written SVP utilized allowed for students to make sense of these dimensions and their interplay at different times and in different ways. While fun and social interaction appeared frequently in students' reflections and discussions, we see also how the SVP facilitated students to consider their experiences in relation to cognitive and physical learning when considering the features of challenge and motor competency. Vital to this was the facilitation of reflection and group processing before, during, and after tasks. Again, the opportunity for students to critique, reflect, and ascribe meaning to their physical activity experiences inside and outside of PE provided them with a guide through which to shape future PE, physical activity, and movement experiences. Echoing previous work by Ennis (2017), and drawn upon more recently by Ní Chróinín et al. (2021) in their work with primary/middle school level students, findings here also emphasize the need for teachers to continuously assist “students in their search to find meaningful experiences in which they seek to engage and affiliate with others in an enjoyable physical activity environment” (Ennis 2017, p. 248). We see how the SVP engaged students in a deep and continuous process of assessment for learning, culminating in a deeper sense of what a meaningful PE and physical activity experience was for them, and the significant role SEL played in this pursuit. In this way, students' ODR marked the departure point for their continued pursuit of MPE rather than the end.

## Conclusion

This study highlights the significance of enacting SVP deliberately and consistently, with findings demonstrating how doing so can lead to the development of SEL and MPE experiences complimenting multiple domains. Crucially, we see here that enacting student voice is an innately social and affective learning process—the latter being something the subject and practitioners have historically struggled to accomplish (Bailey et al., 2009; Dyson, 2014; Dyson et al., 2020; Wright and Richards, 2021). We need to find better ways of consistently listening and responding to students in PE that are also feasible for teachers to implement, and we also need to provide better parity for SEL. Of great lament, is that this was the first-time students had engaged in such a process in PE. We approached this class with students utilizing simple practices to enact SVP explicitly prioritizing SEL and MPE to intentionally facilitate a more holistic learning experience as well as a means through which to assess subsequent student learning. Future work must look to further bridge the gap between enacting and drawing on the voices of the students we work with, and providing them with the agency and space to make responsible decisions around their participation in physical activity and movement on their own and with others, inside, and outside of class. We encourage practitioners to draw from and utilize some, if not all, of these SVP, and modify them in a practical manner that aligns with their own curricular outcomes that target student voice and SEL. Future research should also look to examine how SVP such as these might serve as potentially useful formal assessment tools to ensure such outcomes are being met and can help create a language and routine around reflection and decision-making which is often lacking in PE settings. We see from this study how the benefits of drawing on the prior and current knowledge and emotional experiences of students and doing so in a continuously democratic and reflective manner can help inform and influence current and future learning. If youth/student voice is to be authentically enacted, it requires everyone's authentic attention—students, teachers, policymakers, and researchers.

This is the first time we have attempted to combine these SVP all at once. If the field is serious about providing students with *voice and choice*, then it is important to be transparent about what this looks like in practice—it is an intricate and fluid process. So too must we better consider how we enact *choice of voice* also when seeking to work more democratically with students through multiple pedagogies and research methods similar to those we have presented here. We encourage both practitioners and researchers in future to utilize these SVP as starting blocks rather than end points in enacting student voice. As pointed out by one of the reviewers, fidelity is not really at the heart of this kind of work, more so a continuous pursuit of sound pedagogical decision making that is accessible and understandable to students and responsive to their voices, needs and abilities. We especially recommend further embedding and exploration of SVP within early years and elementary education, where such pedagogies are especially lacking (Iannucci and Parker, 2021), with a view to their continued refinement through to adolescence.

In implementing and researching these SVP all at once, we were concerned from the outset as researchers and practitioners that we may have been *changing too much too quickly* and moving away from what might be considered *conventional PE*. What subsequently occurred suggests the change in approach to enact student voice to prioritize MPE and SEL was welcomed and embraced. We call for further embedding of SVP capturing students' physical activity and movement experiences inside and outside of PE in teacher education and professional learning and development that helps teachers, and their students, make sense of, shape, influence, and enact more meaningful PE and physical activity learning experiences. In closing, we consider the final words of Auria as to why:

“If you want me to be honest with you, at first, I was like ‘This is stupid…you don't do work in gym'. Cause' I was never used to getting to express myself in gym or I've always been used to just actually like testing, like on your physical ability. Yeah, so now that I look back at it, I like it because we get to express ourselves. And, instead of testing on our, like, physical ability, you're kind of looking back at people's mental ability and seeing what they like and what they don't like. And maybe like, say for instance, you're asking us what we're struggling with and why and what we want to continue. So, I felt like it was really good. But at first, I was like ‘This is not gym”' (Auria, FGB).

## Data Availability Statement

The datasets presented in this article are not readily available because all data is stored safely using University online depository. Requests to access the datasets should be directed to dfhowley@uncg.edu.

## Ethics Statement

The studies involving human participants were reviewed and approved by Office of Research Integrity, University of North Carolina at Greensboro, 2718 Moore Humanities and Research Administration, 1111 Spring Garden St., Greensboro, NC 27412, Study Reference #21-0312. Written informed consent to participate in this study was provided by the participants' legal guardian/next of kin and participants.

## Author Contributions

The first author claims first authorship of the work, has made substantial, direct, and intellectual contributions to the work, and approved it for publication. All other authors have made additional minor, direct, and intellectual contributions to the work, and approved it for publication.

## Conflict of Interest

The authors declare that the research was conducted in the absence of any commercial or financial relationships that could be construed as a potential conflict of interest.

## Publisher's Note

All claims expressed in this article are solely those of the authors and do not necessarily represent those of their affiliated organizations, or those of the publisher, the editors and the reviewers. Any product that may be evaluated in this article, or claim that may be made by its manufacturer, is not guaranteed or endorsed by the publisher.

## Footnotes

^1^United States Department of Education. *Race to the Top District Competitions Draft Definitions*. Available online at: https://www.ed.gov/racetop/districtcompetition/definitions.
