# Supplementary material for: “This Is Not Gym”: Enacting Student Voice Pedagogies to Promote Social and Emotional Learning and Meaningful Physical Education
Source: Front Sports Act Living. 2021 Oct 26;3:764613. doi: 10.3389/fspor.2021.764613 (PMC8576448; doi:10.3389/fspor.2021.764613)
Supplement: Supplementary file 1 [file Data_Sheet_1.pdf]

Supplementary File

Expanded Detail on Enacted pedagogies drawn from literature and research prioritizing Student Voice

Week(s)	Enacted pedagogy drawn from literature and research prioritizing YSLV	Details of Enactment within this Study
1-2	Full Value Contract<br>(Tannehill & Dillon, 2009)	The teacher presented their rules, routines and expectations RRE's with students at the beginning of the course. Students were then invited to modify and provide additional RRE's for subsequent PE lessons.
1-2	Personal Biography<br>(Betourne and Richards, 2015; Sutherland and Parker, 2020)	Students completed a personal biography to help them reflect and share their previous life experiences and relationship with PE and physical activity with their peers in class, guided by the following three questions:<br>• What was your experience growing up in your family group, school community?<br>• What do you feel is your social identity and personal identity?<br>• How do you think these experiences have shaped your relationship with physical education and physical activity?
1-10	Cooperative Learning and Group Processing<br>(Dyson and Casey, 2012, 2016; Sutherland et al., 2019)	Cooperative Learning was used as the primary pedagogical method during Weeks 1-4 when the focus was on building relationship skills with students in class. Structures implemented were Learning Teams, Jig-Saw, Think-Pair-Perform, Rally Round Robin. Cooperative Learning structures were frequently used throughout the remainder of the course at different times, but alongside other instructional pedagogical practices such as peer tutors, task stations, direct instruction, intra-task variation, mastery learning, play-teach-play, and child designed activities (Graham, 2008). Particular emphasis was placed throughout the course on the cooperative element of group processing. Students regularly engaged in group processing during and after tasks throughout the course (e.g. *What happened? So what? Now What?*). This occurred in pairs and/or small groups who then shared their discussions with the teacher and class to help inform future lessons and planning. The Learning Team structure was implemented during the final unit of work (floor hockey) when students were invited in heterogenous groups to *invent a game* incorporating the basic rules and skills of floor hockey which they learned, performed, and taught to other groups while reciprocally learning and performing theirs.

2-10	Continuous Class Consultation & Negotiation<br>(Enright and O'Sullivan, 2010a; 2010b; Howley and Tannehill, 2014; Howley and O'Sullivan, 2020, 2021; Aarskog et al., 2021)	Outside of the formal methods listed, students were continuously encouraged to reflect on and discuss their experiences of PE, critiquing them, and providing feedback before, during and after classes to inform the planning, content, learning and assessment through opportunities for: discussion; questioning; verbal feedback; taking quick votes and debriefing. Students engaged in regular dialogue and discussion on PE experiences to negotiate curriculum and lesson content outside of the methods listed here. This was especially conducted during the facilitation group processing when using Cooperative Learning, which required the shared reflection and planned action of students and teachers going forward (*What happened? So what? Now What?*). At different times, students were asked to appraise their experiences after lessons using verbal and written feedback to help inform future planning and these were collected as artefacts. In Week 8 following the final taster sessions, the teacher and students discussed and negotiated what unit of work to pursue based on the activities they selected in the taster sessions. After agreeing to select floor hockey, they discussed how it could be meaningfully taught in lessons through a collaborative reflection of their experiences up to that point, outlining group expectations reflecting a commitment to FVC and the oncoming lessons. Within the unit of work, students then worked their way through the content, eventually designing modified games which they learned, performed, and taught to other groups. while reciprocally learning and performing theirs.
3-4	Timeline<br>(Enright and O'Sullivan, 2012b)	Students created a personal timeline identifying a minimum of 5 or a maximum of 10 significant moments in their life that impacted on their health and wellness, be they positive or negative experiences. In doing so, they were asked to provide (a) some detail about each event (b) the impact they think it had on their relationship with physical activity (d) how they responded (c) what they learned from each event. They then shared some of these experiences with others in class.
4-7	Taster Sessions<br>(Enright and O'Sullivan, 2010a; 2010b; Howley and Tannehill, 2014)	In Week 4, students were presented with a grouped list of movement activities. Students were asked to vote on their preferred two activities from each group (Selected activities highlighted in bold). They were also invited to suggest two more not on the list (Students' Call). They then participated in a 30-minute taster of the class' selected movement activities with a view to pursuing one as a full unit of work.<br>Group 1: **Meditation, Yoga**, **Barre**, Gymnastics

		Group 2: **Tag Rugby, Ultimate Frisbee**, Quidditch<br>Group 3: **Spikeball, Pickleball,** Badminton<br>Group 4: **Floor Hockey, Orienteering**, Rounders, Golf<br>Students' Call: **Basketball, Soccer.**<br>These highlighted activities were then later voted on and discussed by students and the teacher when negotiating and selecting the final unit of work to implement (See Continuous Class Consultation and Negotiation).
7-10	Photovoice Task 1<br>(Enright and O'Sullivan, 2012b; Azzarito and Kirk, 2013)	Students were invited to take a minimum of 5 photos based on the following five prompts: 1) Where I spend my leisure time; 2) my physically active life; 3) physical activity facilities nearby; 4) physical activity in the lives of my family and friends; and 5) the things that are important to me. These photos could not include human subjects. Students were then asked to explain what each photo represented in one sentence. They were invited to orally share/explain their selection of pictures with peers class.
9-10	Photovoice Task 2<br>(Enright and O'Sullivan, 2012b; Azzarito and Kirk, 2013)	Students were invited to seek out and describe a "meaningful" experience in relation to PE and/or physical activity outside of class (i.e. one that is fun, involves social interaction, challenge, motor competence, and personally relevant). They were invited to take a maximum of 5 photos that helped them describe and represent the activity as they experience it. (e.g. take photos of the space where you perform this activity, or the equipment you use). They could not include human subjects in the photos. Students were then asked to explain why this movement activity was meaningful for using the following prompts:<br>Explain how/why/or if it involves: 1) fun; 2) social interaction; 3) challenge; 4) motor competence; and 5) personally relevant learning.<br>They were asked to begin each section with the following statement "I find this..." (e.g., I find this fun because; I find this socially interactive because). Finally, they were asked to think about how this meaningful PE/physical activity experience they presented and described contributed to their SEL development? (e.g. Does it help them to self-manage, be self-aware, socially aware, develop relationship skills, and make responsible decisions?
2-10	Digital Reflections<br>(Lynch and Sargent, 2020; Sargent and Lynch, 2021)	At the end of classes each week, students were asked asked to take some time to think back on their learning experiences in the previous two classes, reflect on these

		experiences and the experiences of those around them. The following questions were used as prompts to help guide reflections.<br>• What happened?<br>• What kind of actions and movements were you performing?<br>• What kind of emotions and thoughts were you thinking about/experiencing?<br>• How do you think it felt for others?<br>• What challenges did you face?<br>• What have you learned from these two classes?<br>• What would you like to see more/less of in the next class?<br>• Any other thoughts?<br>These reflections were submitted digitally in written and recorded form. Students were invited to submit using other ideas other formats (e.g., drawing, poem, song, etc.) but chose not to.
10	Overall Digital Reflection (Lynch and Sargent, 2020; Sargent and Lynch, 2021)	Based on their learning experiences inside and outside of the class, students asked to create a *proclamation of meaningfulness* describing the types of emotions, experiences, and movements involving physical education/activity that were meaningful to them and why. They were asked to complete the following five statements:<br>1 - I've learned that physical education and physical activity is fun when....<br>2 - I've learned that physical education and physical activity is socially interactive when....<br>3- I've learned that physical education and physical activity is challenging when....<br>4 - I've learned that physical education and physical activity improves my motor competence when...<br>5 - I've learned that physical education and physical activity is personally relevant to me when...<br>Finally, they were invited to consider and explain how to make these emotions, experiences, and movements a regular part of their day-to-day life and in the future.
